# Role-theoretic discourse analysis of German security policy: a case of German parliamentary debate on the mission in Afghanistan

**DOI:** 10.3389/fpsyg.2023.1329151

**Published:** 2024-01-09

**Authors:** Xiaoshan Ni

**Affiliations:** College of Foreign Languages, University of Shanghai for Science and Technology, Shanghai, China

**Keywords:** role theory, linguistic discourse analysis, topos/topoi, security policy, military operations abroad

## Abstract

This article combines linguistic discourse analysis with role theory to create a role-theoretic discourse analysis framework for German security policy. To illustrate this, we employ topos analysis on 30 plenary minutes of parliamentary debates regarding the International Security Assistance Force mission in Afghanistan, conducted by German Members of Parliament between 2001 and 2014. We interpret their perception of Germany’s roles in light of key behavioral norms related to German security policy. The parliamentary discourse, shaped by topoi, sets the stage for decisions on German military operations abroad. The use of topoi is influenced by dominant thought patterns, particularly the perspective on Germany’s role in security policy held by the political elite. Political decisions, in turn, reflect behavioral preferences guided by these viewpoints. Our research reveals how changes in German security policy are mirrored in the discourse. This discourse is structured around five categories of topoi for legitimizing or delegitimizing military operations abroad: necessity, obligation, self-interest, capability and preparedness, and solution. An evaluation of the use of topoi through the lens of role theory indicates that perceptions of Germany’s role have evolved over time, encompassing roles such as a “civilian power,” a “normal state,” and an “agenda-setting role” in sync with its foreign military engagements.

## 1 Introduction

Since 1990, extensive research has been dedicated to examining German foreign policy, with a particular emphasis on Germany’s role in diplomatic endeavors. In the traditional realm of international relations, security policy is considered an integral component of foreign policy (Hellmann, 2007 p. 605). Following German unification, the nation’s security policy underwent a notable transformation characterized by a “proactive” strategy that prioritized “crisis prevention” and “long-range defense.” By the mid-1990s, this approach increasingly incorporated the use of military force as a measure of last resort (Hellmann, 2007 p. 612). Within this context, the deployment of the Bundeswehr to Afghanistan held significant importance (Li, 2010 p. 4). The Bundeswehr’s involvement in Afghanistan as part of the International Security Assistance Force (ISAF) spanned a period of fourteen years. This prompts the question: has German security policy evolved as a consequence of this process? This inquiry has been extensively explored in an abundance of scholarly work, employing a range of political science theories and methodologies (Brummer and Fröhlich, 2011; Noetzel, 2011; Hilpert, 2014). During the last Bundestag debate on the Bundeswehr’s participation in ISAF, which took place on 13 February 2014, member of parliament (MP) Annen (Social Democratic Party, SPD) remarked, “The Bundeswehr mission has also altered our nation and our political discourse.” This paper therefore attempts to construct an analytical framework to scrutinize the development of German security policy through the lens of language.

Prominent international political scholars such as Ashley (1987), Walker (1987), and Shapiro (1988) have already underscored the significance of the “power of language” within the realm of international relations. Drawing upon theories from post-structuralist philosophers, they have recognized that “discourse” serves as a linguistic system for generating meaning (Foucault, 1974). Through this lens, they have conceptualized foreign policy as a “discursive practice” and harnessed discourse theories to provide critical analyses of how states and international institutions construct their foreign policies (Hansen, 2016 p. 95). Milliken (1999) has contributed a valuable introduction to discourse analysis and its underlying theoretical principles. Notably, this work represents one of the early instances of explicit discussions on discourse analytical methodologies. Waever (2003) has presented his widely accepted theory on European integration policies, conceiving them as layered discourses. Furthermore, he has explored the intersection of discourse analysis with the field of foreign policy analysis. Hansen (2016) has positioned post-structuralist discourse analysis within the landscape of international relations, offering methodological guidelines on how to read and select texts and construct research designs. This approach effectively highlights the prevailing representations of social reality and the diverse interpretations of it, as illustrated by post-structuralist discourse theory. However, research employing this methodology sometimes neglects the crucial “linguistic” dimension involved in the creation of subject identities (Aydın-Düzgit, 2014 pp. 355–356).

Linguists like Brown and Yule (1983) adopt a predominantly linguistic perspective for the analysis of discourse. Fairclough (1992) contends that systematic textual analysis forms an integral component of linguistic discourse analysis (LDA). Jensen posits that LDA facilitates the observation of communicators’ construction of semantic networks, which reflect their worldviews and elucidate the process of developing a shared understanding (Hacker, 1996 p. 38). Starting from the 2000s, anthologies by Barton (2003), Warnke (2007), Felder et al. (2011), as well as contributions by Busse and Teubert (2013b), and textbooks authored by Niehr (2014) and Bendel Larcher (2015), have collectively contributed to the crystallization of LDA. Linguists regard LDA as an extension of text linguistics, acknowledging that discourse, as a linguistic unit, transcends the boundaries of individual texts (Niehr, 2014 p. 29). LDA, functioning as a descriptive analytical approach, is employed to characterize the distribution, meaning production, and knowledge construction of linguistic phenomena within discourse. It should be noted that the primary aim is not to evaluate or interpret these phenomena (Bendel Larcher, 2015 p. 37). The Düsseldorf School, which builds on the theory of historical semantics (Spieß, 2011 p. 88), has made significant strides in the development of linguistic discourse theory (Spieß, 2011 p. 107). Their work includes extensive empirical investigations of political discourse and the development of linguistic discourse analysis on three key levels: lexical, metaphorical, and argumentative (Wengeler and Böke, 1997; Wengeler, 2003; Jung, 2013; Stötzel and Wengeler, 2013; Niehr, 2014). While some have critiqued the perceived lack of societal relevance in mere descriptions of language usage, it’s essential to recognize that descriptive analysis, with its capacity to deconstruct and reconstruct discourse, can offer valuable insights (Bendel Larcher, 2015 p. 222). It is precisely in this context that interpretive theories in the domains of international relations and political science can offer pathways to comprehending the political connotations inherent in discourse. Post-structuralist research has underscored the pivotal role of discourse in the field of international relations (Hansen, 2016), offering a robust explanatory framework for issues related to security policy.

This study draws inspiration from Schünemann et al. (2018), who employ role attribution to elucidate the political conduct of state actors, asserting that role attribution manifests in the keywords used in government documents. To establish a hybrid approach that amalgamates the descriptive and explanatory capabilities of discourse analysis, we connect role theory (RT) in international relations (IR) research with LDA tools (operating at the argumentative level). This fusion results in a role-theoretic discourse analysis framework for examining German security policy. In order to exemplify the application of this role-theoretic discourse analysis framework in the context of security policy research, we conduct an empirical discourse study on the German parliamentary debate on ISAF. This study seeks to address the following research questions: How did changes in Germany’s security policy throughout the fourteen-year ISAF mandate, spanning two Gerhard Schröder administrations and three Angela Merkel governments, manifest within the parliamentary discourse on ISAF? What shifts in Germany’s role in security policy do these discourses signify?

## 2 Research theory and methodology

### 2.1 Role theory and study of German security policy

Role theory originated in the 1930s with its foundation by sociologist Mead, primarily as a framework to elucidate human behavior (Breuning, 2017). Subsequently, Holsti (1970) extended RT’s application to the realm of international relations, using it to expound upon the varied patterns of foreign policy conduct. Throughout the 1970s, RT underwent further development and emerged as a theoretical paradigm for the examination of comparative foreign policy (Walker, 1987). It is widely acknowledged that RT serves as a valuable analytical variable for investigating the motivations underlying state actions, grasping the perspectives of state actors, and predicting their diplomatic behaviors (Yuan, 2013). According to Harnisch (2015), a state’s role can be parsed into two components: the Ego-part and the Alter-part. The Ego-part encompasses the inherent perception of national interests by decision-makers, reflecting domestic pressures and considerations. Conversely, the Alter-part represents another facet of decision-makers’ perception, encapsulating the expectations of other actors within the international community. Foreign policy-related roles are significantly influenced by the expectations of both the domestic departments (ego-expectations) and international counterparts (alter-expectations), playing a pivotal role in shaping and guiding foreign policy behavior (Harnisch, 2011 p. 8). The configuration of Germany’s security role is intricately intertwined with the parliamentary discourse surrounding the legitimization or delegitimization of military engagements abroad. The process of this political discourse serves as a mirror, reflecting the perception, establishment, and reshaping of Germany’s role by the nation’s political decision-makers. Given this context, RT emerges as an apt explanatory framework for understanding how Germany’s security role is articulated within the political discourse on security policy. Numerous scholarly contributions have delved into the positioning of Germany’s role within the post-Cold War international system. This study has specifically identified “civilian power,” “normal state,” and “agenda-setting role” as representative role prototypes. These prototypes have been selected as the foundation for a qualitative analysis grounded in RT.

The theory of “civil power” as articulated by Maull (1989, 2000) has been frequently employed in the evaluation of post-unification Germany. “Civilian power” denotes a category of actors that intentionally differentiate themselves from “traditional powers” regarding their objectives and strategies in contributing to the evolution of international relations. Their overarching aim is to promote the advancement of international political civilization, and they exhibit a distinct foreign policy value orientation and style (Kirste and Maull, 1996). The selection of “civilian power” as the archetype for Germany’s security role in this study implies that Germany assumes a relatively “demilitarized” character compared to other nations. Furthermore, it conveys that the goals and methods of German foreign policy possess a “civil” quality and are significantly influenced by Germany’s “culture of restraint” (Meiers, 1995). Identifying Germany as a “civilian power” does not signify the complete absence of elements related to “power” and “material interests” in German security policy; rather, these aspects must be assessed, deliberated upon, and balanced through a framework of norms and values inherent to the role prototype of a “civilian power” (Harnisch, 2001).

The concept of the “normal state” as used in this article originates from Germany’s foreign policy strategy of “normalization”, in contrast to the “de-normalization” approach followed by the Federal Republic of Germany during the Cold War. “Normality” emerged as one of the most frequently used terms following German reunification (Walser, 1998). It is the legacy of fascism in Germany’s history that challenges this pursuit of “normality” (Wiegel, 2001 p. 28). According to the linguist Assmann and Frevert (1999), “normality” encompasses various interpretations, including the abandonment of the German “Sonderweg,” the reclamation of national sovereignty, the termination of occupation, and the removal of political constraints. Authors such as Baring et al. (1991), Schöllgen (1993), and Schwarz (1994) have explored “what Germany’s new role should entail” in their publications, and they unanimously concur that the past should no longer impede the present. In his book *Der deutsche Weg* (*The German Way*), SPD politician Barr advocates for a German foreign policy liberated from its excessive preoccupation with the past (Barr, 2003 p. 137). With the Federal Constitutional Court’s landmark decision in 1994 permitting the Bundeswehr to engage in extraterritorial military operations, a growing number of observers have asserted that Germany has transitioned into a “normal state,” free from historical constraints and capable of employing force when necessary (Baumann and Hellmann, 2001). As Germany’s influence has grown, the international community has come to anticipate that it will assume a “normal” international role akin to that of Britain and France, actively participating in international affairs beyond the economic realm (Brummer and Oppermann, 2015).

The third representative role prototype is the “agenda-setting role.” In international regimes, the agenda is typically established, or jointly determined, by the Great Powers in alignment with their national interests and value standards. For example, Nye (1990) highlights the United States as a paramount power source in the international system and a crucial component of soft power. By incorporating its ideals into the expansion of the international system, the United States has gradually forged a set of exceptionally legitimate and stable competitive norms. Germany’s commitment to the principles of multilateralism, its proactive utilization of its power and resources to enhance its influence, and its increasingly active participation in global organizations such as the European Union (EU) and North Atlantic Treaty Organization (NATO) have been recognized as indicators of its shift toward an “agenda-setting role” (Bulmer and Paterson, 1996; Anderson, 1999). A study by Rittberger’s team on German foreign policy after reunification revealed Germany’s insistence on delegating authority and responsibilities to supranational and international organizations, even expanding its involvement in various domains (Rittberger, 2001). Becoming a “leader” akin to the United States epitomizes the “agenda-setting role.” However, contingent upon the level of influence, it can also assume the role of a “follower with agenda-setting function” (Cooper et al., 1991) or a hybrid role within the “leader” framework, known as a “broker” (Maull, 2008).

These three role prototypes were selected for their relevance to Germany’s post-unification security policy, encapsulating the nation’s stance in multilateral international relations and its approach to addressing global security concerns through overseas military interventions. While not mutually exclusive, they do entail distinct behavioral norms. In our empirical study, we can categorize the key behavioral norms associated with each prototype, highlighting these distinctions as assessment indicators for evaluating Members of Parliament’s role perceptions during parliamentary debates (see Table 1).

**TABLE 1:** Representative role prototypes of Germany related to security policy.

Role prototypes	Key behavioral norms
CIVILIAN POWER	– Be values-oriented, – Emphasizing the rule of law in international relations, – Willing to assist, – Accepting collective discipline and valuing cooperation and solidarity. – Preference for non-violent means of conflict solution.
NORMAL STATE	– Be national interests- oriented, – Be vulnerable to external expectations, – Emphasizing responsibility and power, – Be relieved of historical constraints and able to use force if necessary. – Adheres to Western alliances and consolidates the trust of allies.
AGENDA-SETTING ROLE	– To seek international influence as a core strategic goal, – Even as a follower, one should strive to play the agenda-setting function, – Make good use of the broker status, – Be an agenda-maker, the absolute leader.

### 2.2 Discourse, linguistic discourse analysis and topos analysis

The modern concept of “discourse” traces its roots to Foucault, who defined it as a network of statements within a society during a specific historical period, guided by “structures of order.” These structures, often implicitly, dictate how individuals can discuss, or not discuss, various aspects of the world in the sciences and public life of that era (Bendel Larcher, 2015 p. 20). This perspective has fostered interpretations within disciplines like philosophy, history, sociology, and political science. The interdisciplinary nature of discourse opens the door to integrating the study of international relations with LDA. Discourse can serve as both a “linguistic unit” and a “practice.”

In the sociological paradigm, discourse encompasses both a communicative act and a social practice that constructs reality and knowledge. Social actors within the discourse assume specific roles, sometimes forming implicit alliances with unequal resources for articulation and resonance generation (Keller, 2011 p. 67). The terms “discourse” and “discursive practice” are interchangeable in this context. The parliamentary debate on ISAF serves as the research discourse, connecting MPs’ role perceptions and operational preferences in an argumentative chain of cause and effect. Military operations are guided by socially constructed beliefs, values, and perceptions that evolve through discursive practice (Schünemann et al., 2018). This research discourse possesses distinctive social characteristics: (1) It exhibits a national trait: parliamentary debates play a pivotal role in shaping the political elite’s perception of the state’s role and serve as a platform for the direct expression of values and perspectives about the state. (2) It conveys group positions: MPs employ pro-arguments to uphold their party or parliamentary group’s stance and anti-arguments to challenge opposing parties. (3) It mirrors German political culture: The Bundeswehr’s engagement in overseas operations was contingent on NATO or United Nations (UN) approval post the German Federal Constitutional Court’s 1994 ruling. Decisions regarding military deployment must be collectively discussed and pre-approved by the Federal Parliament. Consequently, discourse is a window into the evolution of national security policy and changes in the state’s role. Given the role of parliamentary discourse in identifying and articulating political concerns and interests, analyzing MPs’ recurring linguistic patterns and rhetorical strategies unveils their ideological commitments and argumentation techniques (Ilie, 2015), including their perceptions of the state’s role.

The linguistic definition of discourse provides a methodological foundation for discourse analysis. Discourse, defined as a “corpus of texts” according to Busse and Teubert (2013a), is a widely accepted concept in linguistics. Positioned above the level of individual texts, discourse exhibits thematic relevance, continuity, and a constant state of evolution (Busse and Teubert, 2013a). Chronological discourse analysis allows for the exploration of shifts in meaning through alterations in discourse. LDA serves as a descriptive-analytical approach, enabling the description of texts by examining the distribution of linguistic elements, meaning production, and knowledge construction within discourse (Berry, 2014; Niehr, 2014). In the realm of international relations, people frequently engage in debates concerning various attitudes, intentions, and behaviors. Parliamentary discussions hold particular significance as a form of argumentative behavior in politics, serving to either legitimize or delegitimize government actions, advocate for political claims, and defend political beliefs and practices (Klein, 2019 p. 65). Interpretive patterns, known as “topos” (plural: topoi), within discourse are closely tied to the collective knowledge and meaning construction of political actors. Therefore, topos analysis of parliamentary debates stands as a valuable tool for the study of security policy discourse.

The term “topos,” much like “discourse,” is multifaceted in its meanings (Knoblauch, 2000). Therefore, it is essential to clarify its application in this context. “Topos” originates from Aristotle’s concept of “Topik,” representing a persuasive mode of thought and argumentative structure. Drawing on the Toulmin argument model (Toulmin, 2003), Wengeler (2003) expounds upon topoi and related concepts, elucidating how discourse analysis can be conducted at the argumentative level. Argumentation is the linguistic process wherein a non-controversial (or less controversial) argument, which can be categorized into pro and contra arguments depending on the position, is employed to either support or counter a more contentious conclusion. The foundation for defining and categorizing topoi is the warrant that establishes the link between arguments and conclusions, ensuring arguments can substantiate those conclusions.

Using the ISAF Mission as an example:

Argumentation 1: Given that the ISAF Mission upholds stability in Afghanistan (Argument 1), it follows that the Bundeswehr should be deployed to Afghanistan (Conclusion 1).
Argumentation 2: In cases where the ISAF Mission results in a deterioration of the situation in Afghanistan (Argument 2), the logical conclusion is that the Bundeswehr should not be sent to Afghanistan (Conclusion 2).

Both argumentations rely on the same warrant–the imperative to support actions that preserve stability, employing a stability topos. In this context, the definition of the stability topos is succinctly expressed in a causal sentence: “The decision to deploy or not deploy the Bundeswehr to Afghanistan is contingent on whether the ISAF Mission can or cannot maintain stability there.”

The term “topos” is employed here as an argumentative-analytical category (Wengeler, 2003 p. 177). According to Horsbøl (2019), a topos serves as a versatile lens enabling analysis at various levels of perceptual distance, ranging from in-depth scrutiny of the linguistic articulation of individual topoi to the typological identification of a set of topoi, and even quantitative analysis of the prevalence and distribution of numerous topoi. The quantitative tracking and tallying of each topos’ occurrences over time seek to unveil the predominance, trends, and ubiquity of specific political thought patterns, as well as their relative significance. Consequently, this approach offers a more comprehensive understanding of discourse history and the political treatment of a subject than can be gleaned through the isolated analysis of individually selected texts or the description of the evolving meanings of isolated words (Wengeler, 2003 p. 297). In empirical research, Wengeler (2003) gauges and compares topoi related to pro- and anti-immigration positions in the German press across different periods to illustrate the evolution of the discourse surrounding German immigration policy. Bauder (2008) conducts a quantitative topos analysis on a dataset comprising five prominent Canadian English-language daily newspapers to identify the most frequently occurring and relatively consistent topoi associated with immigration in media discourse. By utilizing quantitative topos analysis, we can unearth the frequently used topoi both in support of and against the deployment of ISAF, as well as track how these topoi have evolved over time. This data can then be leveraged to assess how the discourse mirrors Germany’s prevailing role and role transitions.

### 2.3 Research design for a role-theoretic discourse analysis

**FIGURE 1:**
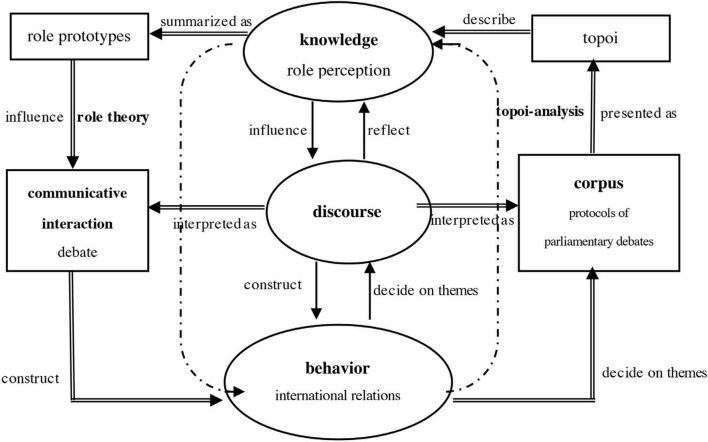
A role-theoretic discourse analysis framework for German security policy.

With a clear understanding of the concepts associated with LDA and RT, the following diagram provides a detailed illustration of how the examination of German security policy can be carried out within an interdisciplinary framework of role-theoretic discourse analysis (Figure 1).

The majority of foreign and security policy research in the field of international relations (IR) typically adopts a “knowledge-behavior” approach (indicated by the dotted line): the knowledge that international actors possess regarding a particular theme influences their behavior, and this behavior, in turn, influences their knowledge. Previous research based on RT has primarily focused on examining the international role assumed by a national actor or organization during specific time periods or in the context of particular international diplomatic events. This examination is typically carried out through logical reasoning or by comparing individual diplomatic documents and behaviors. Some studies have also delved into the interaction between national roles and foreign policy (Wang, 2008; Harnisch, 2011, 2015; Zheng, 2017).

Drawing upon a sociological and linguistic comprehension of discourse, this article proposes a “knowledge-discourse-behavior” approach (represented by a single arrow). In this framework, discourse functions as a bridge: international actors’ knowledge of particular themes shapes their expressions, and their statements collaboratively construct the pertinent discourses. These discourses, in turn, exert an influence on their subsequent behavior. Specific political behaviors often serve distinct purposes, thereby determining the specific themes around which the discourse will revolve. The discourse, in this context, serves as a vehicle for conveying the actors’ knowledge of those themes.

Concretely, this paper will follow the subsequent steps in the study (represented by double-line arrows): (1) Creating a corpus of texts is a fundamental requirement for the linguistic analysis of ISAF discourse. The selection criteria, based on the guidelines from Busse and Teubert (2013a p. 14), have been incorporated in this article. These criteria dictate that the chosen texts should pertain to the selected research subject, in this case, the topic “ISAF.” They should also be limited to specific time periods (2001–2014), geographic areas (Germany), and text types (parliamentary debates). (2) To characterize the ISAF discourse, a collection of topoi is extracted. Initial definitions of these topoi, previously developed from relevant research (Baumann, 2006; Ni and Zheng, 2018), are revised during the evaluation of the ISAF discourse. New topoi are introduced, overly broad topoi are refined, and other initially recorded topoi that do not play a quantitatively or qualitatively significant role are excluded from consideration. Achieving a consistent classification of topoi in the ISAF discourse necessitates this additional round of refinement (as indicated in Table 2). (3) Quantitative analysis of the topoi reveals the predominant topoi within each time period, systematically presenting the prevailing patterns of thought among MPs regarding the actions. (4) In order to investigate the shifting roles reflected in the ISAF discourse, a qualitative examination of the dominant discourse formula is carried out in connection to the debate’s content. This analysis utilizes the typical German role prototypes associated with security policy, as outlined in Table 1.

**TABLE 2:** Categorization of topoi in parliamentary debates regarding the ISAF mission.

Characteristics of topos	Category of topos	Sub-category of topos	Keywords for identification of topos
With the most significant obligatory characteristics	*Obligation topos*	*Responsibility and obligation*	Verpflichtung, Pflicht, verpflichtet, versprechen, zusagen (*obligation*); Verantwortung, verantwortung*, (un)verantwortlich* (*responsibility*) etc.
		*Law and regulation*	(völker)recht, Grundgesetz (*law*); Beschluss, beschließen, Mandat, mandatieren, Resolution (*authorization*) etc.
		*Expectation*	Erwartung, erwarten (*expectation*); Bitte, bitten, Aufforderung, auffordern, Ablehnung, ablehnen, Hoffnung, Kritik, Widerstand (*appeal and request*)
		*Value*	international*/ welt*/ Welt- +Frieden, Freiheit, Sicherheit, Krieg, Gefahr, Stabilität, Demokratie, Menschenrecht, Terror*, Drogen* (*universal value*); Solidarität (*coalition solidarity*) etc.
Drift from an ethic of morality to an ethic of success	*Necessity topos*	*Afghanistan’s interests*	Afghanistan/ afghanisch* + Freiheit, frei*, Stabilität, stabil*, Frieden, fried*, Menschenrecht, Terror*, Sicherheits*, (Bürger)krieg (*security, stability and human rights in Afghanistan*); Wiederaufbau, Entwicklung, Polizei, Armee/Soldat, Rechtsstaatlichkeit, Sourveränität, Übergabe der Verantwortung, Selbstbestimmung (*Afghan state-building, “good governance”, democratization, nomocracy*) etc.
		*Interests of the region*	Pakistan/pakistan*, Region, Krisenregion (*crisis area*) etc.
Most clearly seeking self-interests	*Self-interest topos*	*Germany’s essential interests*	Deutschland/ deutsch* + Interesse, Freiheit/frei*, Stabilität/stabil*, Frieden/fried*, Sicherheit/sicher*, Terror* (*Security, stability and freedom in Germany*) etc.
		*Benefits for Germany*	Deutschland/ deutsch* + Vertrauen, Ruf (Name, Rolle, Bild, Ansehen) (*reputation and credibility of Germany*); Außen-/ Sicherheits-/ Entwicklungspolitik (*benefits for Germany’s foreign, security and development policy*); Einfluss (*Germany’s influence*) etc.
		*Interests of Europe*	Europa/ europäisch* + Interesse, Freiheit/frei*, Stabilität/stabil*, Frieden/fried*, Sicherheit/sicher*, Terror * (*Security, stability and freedom in Europe*) etc.
Focus on actionability of actions	*Capability and preparedness-topos*	*Capability*	erfüllen, in der Lage, können, gerecht, Fähigkeit (*capability)*etc.
		*Preparedness*	Weiter-so Politik, bereit sein (*preparedness*) etc.
Direct statements on the use of military force	*Solution topos*	*Military*	militärisch* Mittel, Trupp*, Soldat*, Krieg*, zivil* (*military force*) etc.
		*Non-military*

## 3 Topos analysis

### 3.1 Description of data

Following each plenary meeting, a draft stenographic report is generated. This report is presented to each speaker for review and signature before being archived in the parliamentary records as the Plenarprotokoll, along with a complete audio recording of the entire plenary session. The plenary minutes of parliamentary debates are readily accessible on the official website of the German Federal Parliament,^1^ serving as a reliable and credible source. The 14-year ISAF Mission (2001–2014) saw the leadership of two generations of leaders, including Gerhard Schröder (Schröder I: 1998–2002, Schröder II: 2002–2005), and Angela Merkel (Merkel I: 2005–2009, Merkel II: 2009–2013, Merkel III: 2013–2017). A total of 18 proposals concerning the involvement and continuous participation of the Bundeswehr in the ISAF Mission were presented, leading to 30 debates among the MPs. These 30 plenary minutes constitute the research corpus. To analyze the evolution of discourse over time, we divided the corpus into segments for the Schröder (I, II) and Merkel (I, II, III) administrations. MAXQDA, a computer-based mixed-methods data analysis software, was employed for labeling, coding, and categorizing recurring topoi in the parliamentary debates. The final classification of topoi in the parliamentary debate on ISAF (Table 2) was achieved through iterative manual readings and regular cross-validation. This software facilitates the visualization of trends and unique aspects in quantitative analysis and aids in describing and analyzing the usage of specific topoi in qualitative analysis.

Each parliamentary debate consists of both argumentative and non-argumentative segments (Ajjour et al., 2017). During the non-argumentative portions, speakers often express their party’s stance, such as in Leibrecht’s (FDP) statement, “[…] we agree with the German government’s request to extend the ISAF mandate” (Bundestag, 2005). The argumentative sections comprise argumentative segments, which can range from a single sentence to several paragraphs, and these segments feature specific topoi. These topoi are used either to advocate for authorizing ISAF (indicated by a “+” in the database) or to argue against it (marked with “−”). For example:

Argumentative segment 1 (−): “In the past 3–4 years, the number of soldiers has increased dramatically and at the same time violence has increased.”–Paul Schäfer (DIE LINKE), BT PlPr 17/9.
Argumentative segment 2 (+): “It is not only a question of relations with Afghanistan, but it is also a very central European policy issue that Germany is committed to these issues together with its partners. That is why it is very important that the Dutch and the Danes, together with our soldiers, will participate in this United Nations peace mission in Afghanistan, if the Bundestag agrees.”–Joschka Fischer, Federal Minister for Foreign Affairs, BT PlPr 14/210.

From the two argumentations above, we can extract two topoi: one concerning the benefits for Germany and the other about Europe’s interests. Both of these topoi fall under the self-interest category used to justify the ISAF operation. When two sub-categories of topoi appear in a single argumentative segment, each is counted once. If they belong to the same main category of topoi, then that category is counted twice. If an MP repeats the same content to emphasize a point, the recurring sub-category of topoi is no longer counted.

### 3.2 Results of topos analysis

**FIGURE 2:**
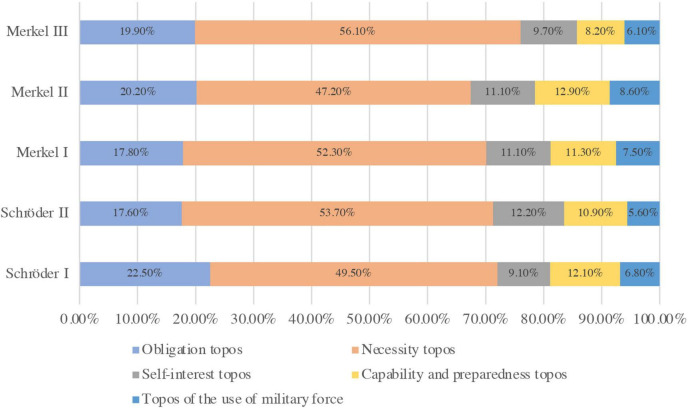
Five categories of topos in the ISAF-discourse. (The frequency of use (%) measures the relative frequency of a specific category of topoi compared to all topoi used during that period.)

According to statistics, MPs used the sub-category of topoi 3,664 times in the ISAF discourse. The (de-)legitimization of ISAF was primarily constructed using five main categories of topoi, ranked by the frequency of use: necessity topos, obligation topos, self-interest topos, capability and preparedness topos, and solution topos (Figure 2). At the macro level, these topoi were used in relatively similar quantities across the periods, as visually demonstrated in Figure 2, indicating a consistent structure in the German parliamentary debate on ISAF over the 14 years. The necessity topos, which prioritizes the interests of the countries and regions involved in the operation, dominated the ISAF discourses under both Schröder and Merkel and tended to increase as the operation progressed. This suggests that German MPs’ views on security policy remained relatively stable throughout this time, consistently emphasizing the “interests of others” rather than “self-interest” in security policy discourse.

Further examination of the subcategories of topoi will uncover the shifts in the German security policy discourse. Table 3 presents the top four to six subcategories of topoi that were most frequently used over time, taking into consideration tied rankings. In contrast to the previously mentioned consistency, these changes occurred at a micro level.

**TABLE 3:** Utilization of sub-categorical topoi in ISAF discourse (Top 4–6).^a^

Periods	Schröder I	Schröder II	Merkel I	Merkel II	Merkel III
Frequency of use of the subcategories of topos	Afghanistan’s interests 47.6	Afghanistan’s interests 51.3	Afghanistan’s interests 49.6	Afghanistan’s interests 41.1	Afghanistan’s interests 54.6
	Value 14	Value 8.8	Value 9.9	Value 11	Value 11.2
	Preparedness 9.8	Benefits for Germany 8.4	Capability 6.3	Preparedness 8.1	Benefits for Germany 7.1
	Benefits for Germany 7.2	Preparedness 6	Benefits for Germany 6.2	Benefits for Germany 6.2	Capability 5.1
		Germany”s essential interests 4.9	Responsibility and obligation 4.2		
			Germany”s essential interests 4.2	

^a^The frequency of use (%) indicates the relative frequency of usage of that specific sub-category of topoi within the specified timeframe, compared to all topoi.

#### 3.2.1 Necessity topos

The primary topos highlighting the interests of the target nation and its surroundings, often employed by MPs to advocate for or against the legitimacy of the ISAF mission, was consistently the necessity topos. At its core, the ISAF mission aimed to aid in state reconstruction within crisis-stricken areas, with the overarching objective of fostering regional stability through support for indigenous security forces and the eventual establishment of long-term defense mechanisms. A specific focus was placed on Afghanistan’s interests, encompassing the creation of a functional state authority, the establishment of a legitimate monopoly over the use of force, and the promotion of democratization processes. These objectives closely aligned with the principles set forth in the Bonn Agreement, which emerged from the International Conference on Afghanistan hosted by Germany in 2001. The fundamental goal of ISAF was to ensure the successful execution of the “Bonn Process.” In the initial stages of ISAF, Defense Minister Struck (SPD) emphasized the necessity for “the protection of an International Security Assistance Force in Kabul and the surrounding area” (Bundestag, 2001). He stressed in the Schröder II that the ongoing stabilization process required reinforcement (Bundestag, 2002a) and that the achieved results necessitated military protection (Bundestag, 2002b). As the operation progressed, concerns arose regarding the major issue of corruption within the Afghan government, particularly its links to the drug trade and warlordism. In response, the Afghanistan Compact was adopted during the Conference on Afghanistan in London in January 2006, shifting the rhetoric among ISAF supporters. This shift emphasized that defending Afghanistan’s interests required increased Afghan accountability (Bundestag, 2008). However, a turning point occurred in 2009 when German officials approved an airstrike near Kunduz, resulting in the unfortunate deaths of over 100 civilians. This incident triggered public outcry both domestically and internationally, prompting a reassessment of the topos related to Afghanistan’s interests. Some argued that the operation was detrimental to Afghanistan’s interests, as it fueled resistance and strengthened international terrorist organizations (Bundestag, 2011b). Left-wing advocates demanded the “withdrawal,” asserting that it marked the first instance since 1945 in which the Bundeswehr had participated in an offensive action resulting in significant casualties. The statement, “this incident shows where we have gone astray” reflects their perspective (Bundestag, 2009c). As the withdrawal date approached, the discourse took on a retrospective and contemplative tone. MPs acknowledged the persistent challenges in nation-building within Afghanistan, while also recognizing the importance of acknowledging the progress made by foreign forces and advocating for ongoing support in the aftermath. Proponents contended that the extended military commitment aimed to facilitate a successful transition of security responsibilities to Afghan security forces (Bundestag, 2014a). In contrast, the opposition characterized the mission as a continued state of occupation and conflict that had claimed a significant number of lives while leaving Afghanistan among the world’s poorest nations (Bundestag, 2014b).

#### 3.2.2 Obligation topos

In terms of usage, the obligation topos takes second place in prominence. The discourse revolving around values, in particular, exemplifies this category. The utilization of the value topos to support Germany’s participation in ISAF underscores the values-driven nature of foreign policy during Schröder’s era. MPs contended that Germany’s involvement in ISAF, geared toward addressing Afghanistan’s humanitarian needs, combating international terrorism, and upholding peace, security, and stability in target nations and crisis areas, stemmed from a commitment to safeguard universal values. In the opening debate, Irmer (FDP) asserted that deploying troops represented “a small stride toward global peace” (Bundestag, 2001). In response to critics arguing that ISAF “undermined human rights,” Schröder argued that Afghanistan exemplified the need for military force to protect innocent people from perpetual suffering (Bundestag, 2001). This perspective remained consistent during Schröder’s second term, emphasizing the pivotal role of the security component. Nevertheless, from Merkel I onward, an increasing number of MPs used the value topos (specifically human rights) to challenge ISAF, citing concerns such as the humanitarian fallout of air strikes or the risks associated with deploying early warning and reconnaissance aircraft. For instance, Lafontaine (DIE LINKE) emphasized the right to life, stating that people must first live before any rights can be ascribed to them (Bundestag, 2007). The presidential elections of 2009 and parliamentary elections of 2010 exposed instances of fraud and abuse of power within the Afghan government, diverging from the initial objective of establishing a democratic Afghan government. Opponents argued that democratization should be an indigenous process and cannot be directly imposed by external forces through coercive means. In Merkel II, the usage of the obligation topos increased in frequency. This was driven by debates on whether actions could truly uphold values and the heightened scrutiny and opposition of operational mandates. MPs stressed that Germany’s participation in ISAF fulfilled security responsibilities and obligations under multilateralism. Defense Minister Guttenberg argued that Germany’s responsibility stemmed from its coalition obligations (Bundestag, 2009b), while Federal Minister for Foreign Affairs Westerwelle stated that deployment was an expression of their alliance solidarity with NATO and the Afghan people (Bundestag, 2011a). The debate surrounding values (particularly human rights) persisted until the final stage. While the opposition decried the operation as “detrimental to the right to life,” “morally shameful, politically wrong, and anti-human” (Bundestag, 2014b), the operation endured, with proponents emphasizing the necessity of enforcing and defending universally recognized human rights, acknowledging the complexity and conflicts inherent in achieving these goals.

#### 3.2.3 Self-interest topos

The self-interest topos and the capacity and preparedness topos, while used with similar frequency, were notably less prevalent than the previous categories. During the Schröder era, MPs believed that military operations aimed to enhance Germany’s reputation in the targeted countries, regions, and among allies. The self-interest topos placed considerable emphasis on the benefits for Germany. Even the opposition Christian Democratic Union/Christian Social Union (CDU/CSU) supported troop deployment, asserting that Germany should “always be a loyal partner in the multilateral ties between the UN and NATO,” according to Pflüger (CDU/CSU) (Bundestag, 2005). Furthermore, Nachtwei (BÜNDNIS 90/DIE GRÜNEN) noted, “[t]he fact that the German government was a driving force in this global effort […] is not a matter of blame but, quite the opposite, is deserving of significant praise. It shows trustworthiness” (Bundestag, 2003). The majority of MPs contended that Germany’s participation in ISAF would not only enhance its credibility and serve its fundamental interests in security, peace, and stability but also yield positive effects on the nation’s economy, politics, and military capabilities. The Federal Minister of Defense in Merkel I emphasized that ISAF was necessary to guarantee stability, reconstruction, and peacemaking, and to ensure the security of German citizens (Bundestag, 2006a). Germany also expanded its influence, taking on lead duties in northern Afghanistan in 2006, assuming the Norwegian Quick Response Force (QRF) in 2008, and initiating the London Conference in 2010. MPs increasingly saw this as a means of enhancing Germany’s influence in the multilateral and international system. The ISAF mission entered an intensified phase during the Merkel administration, marked by a rise in German military casualties and heightened concerns for the nation’s fundamental interests, particularly security. In response to the deteriorating security situation in Afghanistan in 2007, Foreign Minister Jung emphasized the importance of continuing both military and civic engagements (Bundestag, 2006a). In the final phase, questions arose about whether ISAF effectively garnered trust for Germany, and fears emerged about potential damage to Germany’s reputation and the Bundeswehr. Hänsel (DIE LINKE) called for an immediate “complete withdrawal from Afghanistan,” cautioning that failure to do so would perpetuate a “military occupation” and “military intervention,” potentially turning the Bundeswehr into an “occupying force” (Bundestag, 2014b).

#### 3.2.4 Capability and preparedness topos

In comparison to the preparedness topos, which was prominent during the Schröder era, the capability topos gained more traction during the Merkel administration. The use of the preparedness topos reflects the debate over whether Germany is equipped to shoulder international security responsibilities and effectively manage associated risks. Under Schröder’s leadership, the action received broad support among MPs due to the perceived clarity of the UN Security Council’s authority, soldiers’ right to employ self-defense, and the clear separation between the Operation Enduring Freedom (OEF) and ISAF mandates, which excluded participation in violent combat operations. Schröder emphasized the necessity for a robust mandate under Chapter VII of the UN Charter, which he believed provided a sufficient level of self-security and task fulfillment (Bundestag, 2001). Defense Minister Struck further emphasized that the conditions of the mandate facilitated successful task implementation and ensured soldiers’ protection (Bundestag, 2001). In contrast, the use of the capability topos in the Merkel era signified a greater emphasis on Germany’s capacity to assume responsibility and make substantial progress. Germany expanded its military presence by deploying Tornado reconnaissance aircraft and AWACS under the ISAF framework. Defense Minister Jung highlighted the significance of these deployments, especially for air surveillance, coordination, and communication. Furthermore, MPs like Mißfelder (CDU/CSU) cited successful civilian construction efforts, such as infrastructure development, electrification, roads, and water well projects in Afghanistan, demonstrating Germany’s capability to contribute positively to the country’s development (Bundestag, 2009a). During Merkel II, preparedness became more pertinent than capacity as the withdrawal phase neared. It was imperative to establish a detailed and reliable withdrawal strategy. In January 2011, the Federal Government first discussed the possibility of reducing the Bundeswehr’s deployment by the end of the year, contingent on the situation. Federal Minister for Foreign Affairs Westerwelle highlighted the government’s practical approach in Afghanistan, emphasizing realistic goals, means, and a timetable (Bundestag, 2011b).

#### 3.2.5 Solution topos

Although the solution topos, which explicitly conveys views on the use of military or non-military means, wasn’t frequently used in the discourse, the limited argumentative segments highlight a shared sentiment among MPs from both the Schröder and Merkel eras. They generally opposed engaging in violent combat operations, favoring non-military approaches, peaceful conflict resolution, and post-war reconstruction. In Schröder’s administration, Defense Minister Scharping emphasized that military means should be a last resort and play a supporting, not a dominating, role in a comprehensive political process (Bundestag, 2002a). This view was echoed by Federal Minister of Foreign Affairs Fischer, who stressed the importance of an UN-mandated international security force. Under Merkel, Germany’s involvement in Afghanistan aimed to contribute substantially to its development and provide temporary, necessary military protection. Foreign Minister Steinmeier outlined these primary goals (Bundestag, 2006b). During this period, opposition to military action increased significantly among MPs due to the rapid deterioration of the security situation since 2007. For the first time, some MPs expressed opposition to any form of violent force, equating military action with “war.” They argued against a “dual strategy” involving both military and civil efforts, contending that increased development aid and military involvement couldn’t be effectively integrated (Bundestag, 2008). Independent MP Winkelmeier called for an “urgent ceasefire,” contrasting it with what he saw as the “wrong track” pushed by the US (Bundestag, 2008). The controversial actions of Task Force 47 and its opaque deployment, particularly the airstrikes near Kunduz in 2009, reignited debates. MPs expressed concerns that the International Security Assistance Force was adopting a more “warlike” and potentially illegal stance. Schäfer (DIE LINKE) voiced the desire for Germany to discontinue participation in what he deemed the “immoral practice of war,” contrary to international law, whether directly or indirectly (Bundestag, 2012).

## 4 Role theory-based discussion

Germany’s security role and the associated policies are shaped by the values and norms perceived by German MPs, while being inevitably influenced by operational guidelines, laws, and agreements (referred to as the obligation topos). These conceptions of Germany’s security role are a response to the international community and target states’ expectations regarding Germany’s conduct in global affairs, reflecting the necessity topos. Additionally, they are intertwined with Germany’s national interests and strategic objectives (self-interest topos), as well as its capacity and readiness to achieve them (capability and preparedness topos). Concerning the ISAF mission, the opinions of MPs about whether Germany should employ military or non-military means to address issues closely align with the perceived security role that Germany should embrace, falling under the solution topos. The role transitions in the discourse occur at a micro level and are influenced by developments and contingencies within ISAF operations. Depending on the key role-related norms presented in argumentative segments, we will qualitatively examine the segments in which the dominant and representative topoi become evident, establishing the prevailing German security role during different periods. These perspectives shape the behavioral preferences of MPs, which not only directly impact the practical implementation of German security policy but also contribute to the collective knowledge about German security policy. The following section explores the presentation of security policy-related roles in ISAF discourse and highlights the changes that have taken place.

### 4.1 Civilian power

Schröder I marked the initiation of Germany’s involvement in ISAF. During this period, German MPs predominantly advocated for Germany to participate as a “civilian power.” This was evident through recurring themes in their discourse. They emphasized that Germany’s role in ISAF was centered on promoting “good governance,” democratization, and the establishment of the rule of law in Afghanistan. Cooperation, multilateralism, and coalition solidarity were prioritized, along with adherence to international law and support for the UN’s extension and strengthening. They underlined Germany’s willingness to engage in peacekeeping missions while distinguishing between war orders and orders for peacekeeping forces. However, there was limited support for Germany’s efforts to restore its international standing through a more belligerent approach. Under Schröder II, Germany’s position as a “civilian power” remained prominent, with a continued focus on Afghanistan’s interests, particularly democracy building and the training of Afghan security forces. Germany’s active involvement in multilateral operations was a key feature, and its contributions were widely recognized by international partners and the UN. Although the solution topos was not frequently used, it revealed a reluctance to resort to violence, with the majority of MPs supporting Germany’s participation in ISAF for the sake of “collective security” and in accordance with international law. They stressed that the UN-mandated ISAF mission should only be employed as a “last resort” and to maintain peace and stability, as determined by the Bonn Conference. The Merkel administration continued to prioritize Afghan state-building, but there was a growing concern that military action might lead to more casualties among soldiers and civilians. The failure of democracy-building initiatives in Afghanistan highlighted the need to consider regional uniqueness and historical and ethnic aspects, rather than imposing Western ideals. Merkel II witnessed an increase in anti-war sentiment, reflecting Germany’s commitment to the role of a “civilian power” and a preference for non-violent conflict management and resolution. The use of laws and regulations as an obligation topos warned against “warlike” behavior, aligning with MPs’ cautious stance on the use of force. Toward the operation’s conclusion, Germany’s values-based foreign policy came to the forefront, with increased cautions about the potential impact on Germany’s standing as a “civilian power” if these principles were compromised. The evolving security situation in Afghanistan highlighted the importance of combining military and civilian measures, with an emphasis on political solutions rather than relying solely on civilian construction and its military support.

### 4.2 Normal state

While not predominant in Schröder I’s discourse, Germany’s aspiration to function as a “normal state” was already discernible. This inclination emerged due to Germany’s pre-operational efforts, successful promotion of the Bonn Conference, adoption of the Bonn Agreement, and early-stage Bundeswehr performance in Afghanistan. These accomplishments elevated international expectations of German foreign policy, earning respect from Afghanistan and its allies. Nevertheless, Germany lacked the necessary leadership structures, tools, and support systems for effectively managing international activities over extended distances and periods. Schröder II witnessed a more frequent utilization of the self-interest topos, reflecting the escalating importance of Germany acting as a “normal state” aligned with its national interests. This shift was driven by the perceived threat of terrorist counterattacks originating from Afghanistan, necessitating military action to address these security concerns. The emphasis on modern security and defense within multilateral frameworks such as the UN, NATO, the EU, and the Organization for Security and Co-operation in Europe (OSCE) was stressed, aligning with the focus on coalition solidarity to alleviate concerns in neighboring countries about Germany’s “more active” foreign policy. From Merkel I onward, the role of a “normal state” gained prominence in the discourse, accompanied by calls for vigilance against factors that could reverse this trend. This shift was particularly evident in discussions about Germany’s essential interests and the value of coalition solidarity. Military operations increasingly leaned toward a policy oriented around national interests, given the transnational nature of the terrorism threat. The use of military means was seen as necessary to safeguard the security of German military and civilian personnel involved in these actions, and the deployment of reconnaissance and early warning planes was justified by the need to protect German economic interests. Coalition solidarity became a primary means of signaling a readiness to shoulder collective responsibility. Under Merkel II, there was growing consensus to conduct foreign policy in the national interest, with the self-interest topos surfacing more frequently. As the reality in Afghanistan deviated from the initial operational objectives, MPs began to approach their objectives with a more rational and realistic perspective aligned with national interests. The discourse underscored that as Germany actively sought to enhance its standing as a “normal state,” it faced competition with other players in the multilateral system. The final phase of the operation entailed a “responsibility transfer” period, raising concerns about Germany’s long-term capacity to deploy its military abroad and a reevaluation of its policy on extraterritorial military operations. There were fears that Germany’s reputation as a “normal state” could be jeopardized, and the interests of all parties might not be optimally served.

### 4.3 Agenda-setting role

Under Schröder I, Germany garnered international respect for its role as the organizer and facilitator of the UN-led Bonn Conference. It made significant contributions to the national reconstruction of Afghanistan, establishing itself as a proactive participant in shaping international relations and addressing global challenges. However, the concept of “agenda-setting” was not yet fully articulated during this period. Schröder II witnessed an effort to emphasize a “German approach” to collective action, signaling a shift from being a mere “follower” to becoming a proactive actor with an “agenda-setting function.” Germany’s refusal to comply with American demands for increased military investment and its assertive promotion of a military-civilian approach within the multilateral system were attempts to break away from the past pattern of “unconditional obedience” to the US. Starting from Merkel I, the capability topos became more prominent in the discourse, highlighting that ISAF demonstrated Germany’s capacity to address international challenges effectively. Germany took leadership roles in Northern Afghanistan, the EU Police Mission, and supported the broader Afghan mission. In 2008, the Grand Coalition Government initiated discussions on a “gradual withdrawal,” calling on international allies to collaborate on a “responsible withdrawal plan.” This phase was seen as a significant opportunity for Germany to exercise an “agenda-setting function.” In Merkel II, the expanded use of the preparedness topos illustrated Germany’s potential to act as a mediator between Afghanistan and the international community during the withdrawal planning phase. Germany’s publication of the new Afghanistan plan, considered a document of international coordination, preceded the London Conference, which introduced the third pillar of the political process. This showcased Germany’s adeptness in shaping the international agenda. In the final phase, Germany’s achievements in its 14 years of operations were highlighted, emphasizing its capacity to assist target countries in civilian reconstruction and provide military security. Germany’s “in and out” approach with NATO allies demonstrated both its commitment to the alliance and its endeavor to wield greater influence in the multilateral system. Germany aimed to secure an “agenda-setting role” based on its successful experience as an ISAF strategist and facilitator. The German MPs aspired to see Germany become an “agenda-setter” akin to the US by more actively leveraging its resources and power to influence the global system, although this is likely to be a long-term objective.

## 5 Conclusion

This article demonstrates the effectiveness of a role-theoretic discourse analysis approach, using parliamentary debates on the Bundeswehr’s involvement in the ISAF Mission to study German security policy. By adopting a linguistic and sociological interpretation of “discourse,” this framework presents a knowledge-discourse-behavior approach, facilitating a theoretical and methodological connection between RT and LDA. In the empirical analysis, quantitative topos analysis is employed to depict and compare the similarities and differences among the various topoi during different time periods. The results reveal that, despite significant variations at the micro level, the ISAF discourses across these five periods generally adhere to a consistent pattern of argumentation. The collective role-related knowledge, as manifested in the prevalent topoi, is then qualitatively assessed through role prototypes. These findings suggest that Germany’s identity as a “civil power” has remained relatively stable. Germany’s role within the multilateral system has evolved from being a passive responder and follower to a proactive strategist and advocate, particularly during critical junctures. This transformation can be attributed to Germany’s growing strength and the trust and influence it garnered through early initiatives, such as mission commencements, pivotal turning points, and mission conclusions. Especially since the Merkel era, there has been a consensus on the need for the “normalization” of German foreign policy, with a more frequent and overt focus on national interests. Nevertheless, as Germany confronts a “trust crisis” once again, the civilizing attributes of Germany’s role will be emphasized.

Due to space constraints, this paper only adopts the topoi analysis at the argumentative level of linguistic discourse analysis, and does not go into detail on the diversity of approaches at the lexical, metaphorical and argumentative levels. The knowledge-discourse-behavior approach offers several avenues for further exploration. Future research could delve into discourse from both lexical and metaphorical perspectives and draw upon a range of international relations theories to address diverse research inquiries. In addition, although computer-assisted methods and regular communication with native speakers were used to improve the objectivity of the analysis process, manual reading was mainly applied in the definition of the discourse and the extraction of codes, so that the paper is mainly based on logical and subjective analyses and elaborations. In terms of follow-up research, computer technology can be applied more to increase the objectivity of the research.

## Data availability statement

The original contributions presented in this study are included in this article/supplementary material, further inquiries can be directed to the corresponding author.

## Author contributions

XN: Writing – original draft.

## Footnotes

1
www.bundestag.de
